# Association between adverse childhood experiences and fruit and vegetable intake among a national sample of U.S. adults

**DOI:** 10.1016/j.pmedr.2023.102339

**Published:** 2023-07-24

**Authors:** Ivan D. Mendoza, Jorge A. Banda, Zachary Giano, Randolph D. Hubach

**Affiliations:** a Department of Public Health, Purdue University, 812 W State St., West Lafayette, IN 47907, USA; b Department of Biostatistics and Informatics, University of Colorado Anschutz Medical Campus, 13001 East 17th Place, Aurora, CO 80045, USA

**Keywords:** Adverse childhood experiences, Dietary intake, Eating behavior, Life stress, Public health surveillance

## Abstract

Few studies have examined the role adverse childhood experiences (ACEs) have on specific diet patterns. This study assessed the association between ACEs and daily fruit and vegetable intake (FVI). Data were derived from the 2019 Behavioral Risk Factor Surveillance System (BRFSS) which surveys 50 states and three U.S. territories. Participants who completed the ACEs module were included in the analyses (N = 106,967). Total ACEs included the summed responses from the domains of abuse, household challenges, and neglect. FVI was reported by number of times consumed per day. The two fruit items included fruit (fresh, frozen, and canned) and fruit juice. The four vegetable items included leafy greens, fried potatoes, non-fried potatoes, and other vegetables. All fruit and vegetable items were analyzed separately to see which specific items drove the relationship between total ACEs and total FVI, equaling a total of 8 regression models. Every model controlled for poor mental health days, sex, age, ethnicity, income, body mass index, and physical activity. Total ACEs were positively associated with daily intake of fried potatoes (β = 0.008, p =.025), other potatoes (β = 0.008, p =.049), and other vegetables (β = 0.024, p <.001). Total ACEs were negatively associated with daily intake of fruit (β = -0.016, p <.001). ACEs had non-significant relationships with leafy greens and fruit juice. Findings suggests that those with increased ACEs scores report increased consumption of fried potatoes, non-fried potatoes, and other vegetables, and less of fruit. Findings highlight the need for understanding food context and preparation when analyzing the relationship between ACEs and diet intake

## 1 Introduction

The Dietary Guidelines for Americans (DGA) recommends individuals consume core elements including vegetables, fruits, grains, dairy, protein foods, and oils (U.S. Department of Agriculture and National Institutes of Health, 2020). Among these core elements, prevalence data shows daily fruit and vegetable consumption is one of the lowest targets met (Krebs-Smith et al., 2010, Rehm et al., 2016). Adults should aim for 1.5–2 cup equivalents of fruit and 2.5–3.5 cup equivalents of vegetables daily, although exact nutrient needs vary by age, physical activity, and other factors (U.S. Department of Agriculture and National Institutes of Health). Nationally represented data from the 2019 Behavioral Risk Factor Surveillance System (BRFSS) shows that 12% of US adults met daily recommended intake for fruit and 10% met daily recommended intake for vegetables (Lee et al., 2022). This is concerning given medical evidence supports high fruit and vegetable intake (FVI) is associated with a reduced risk of cardiovascular disease, cancer, and all-cause mortality in adults (Aune et al., 2017, Wang et al., 2021). Increased FVI may also decrease blood pressure, (Appel et al., 1997, Fatahi et al., 2018, Yokoyama et al., 2014) improve cholesterol, (Djousse et al., 2004) and stabilize glucose regulation, (Carter et al., 2013) all of which are important biomarkers for the onset of chronic diseases.

Research suggests psychosocial factors are associated with FVI (Sleddens et al., 2015). Stress, for instance, has been a well-documented correlate of eating behavior, (Hill et al., 2022) with research showing an increased stress being associated with caloric intake (Dallman, 2009). For those who consume more in response to stress, food choices made are typically high in fat and sugar; (Torres and Nowson, 2007) both of which are typically not characteristics of nutrient-dense foods such as fruits and vegetables (U.S. Department of Agriculture and National Institutes of Health, 2020). Adverse childhood experiences (ACEs) may be another important correlate to FVI. ACEs are primarily defined as adversity in childhood, varying in severity which occurs in a family/social environment (Kalmakis and Chandler, 2014). People who experience ACEs, like those with a poor diet quality, have been shown to be at a higher risk of coronary heart disease, (Deschênes et al., 2021, Su et al., 2015) type II diabetes, (Deschênes et al., 2018, Huang et al., 2015) and stroke (Wilson et al., 2012).

Toxic stress is one contributing factor for increased morbidity among those with ACEs, (Shonkoff et al., 2012) and it may also describe the relationship between ACEs and FVI. Toxic stress is a unique, prolonged type of stress which has detrimental psychological and physiological effects putting individuals in an increased allostatic load (Shonkoff et al., 2012). An allostatic load is the inability to shut of a stress response after the stressor has been removed, and it has been associated to an unhealthy diet (Guidi et al., 2021). Those in a high-allostatic-load have reported lower vegetable consumption compared to those in a low-allostatic-load (Macit and Acar-Tek, 2020). In response to toxic stress and a high-allostatic-load, people often engage in maladaptive coping strategies including emotional eating. Bouts of emotional eating often accompany hyperpalatable, energy-dense foods, (Camilleri et al., 2014) which may create less opportunities for adequate FVI.

Given a large proportion of adult Americans report to have at least one ACE, (Giano et al., 2020) a growing number of studies have explored the relationship between ACEs and dietary quality (Cammack et al., 2020, Abajobir et al., Oct 2017, Kazmierski et al., 2022). For instance, child maltreatment has been associated with excessive sugar sweetened-beverage intake, (Cammack et al., 2020) fast food consumption, (Cammack et al., 2020) and high dietary fat intake (Abajobir et al., Oct 2017). Higher ACE scores have been related to lower consumption of fruit and vegetables (Horino and Yang, 2021, Yanagi et al., 2020, Aquilina et al., 2021, Windle et al., 2018). After adjusting for sex, age, race, and ethnicity, Windle et al. (Windle et al., 2018) found higher ACE scores to predict lower levels of fruit and vegetable consumption. In a sample of older adults with ACEs, Yanagi and colleagues (Yanagi et al., 2020) found there to be an increased risk of low FVI among both males and females, with higher associations noted among females.

To date, most studies examining ACEs and FVI have grouped fruits and vegetables into a single outcome measurement (Horino and Yang, 2021, Yanagi et al., 2020, Windle et al., 2018). This may limit our understanding of which specific subgroups of fruits and vegetables individuals with high ACEs consume less of. Research suggests subgroups within fruits and vegetables (e.g., dark-leafy green vegetables or citrus fruits) play unique roles on health given fundamental differences in dietary bioactive compounds such as polyphenols and carotenoids (Wallace et al., 2020).

The current aim of this study is to (1) assess the extent to which total ACEs are associated to daily FVI in a nationally representative sample of US adults and (2) to determine which specific fruit and vegetable items drive this relationship. We hypothesize that higher ACE scores will be related to lower consumption of total fruits and lower consumption of total vegetables. Within the total fruits, we hypothesize ACEs will be related to decreased consumption of fruit and increased consumption of fruit juice. Within total vegetables, we hypothesize ACEs would be related to decreased consumption of leafy greens and other vegetables, as well as increased consumption of potatoes and fried potatoes.

## 2 Methods

### 2.1 Data

This cross-sectional study uses public, de-identified data from the Centers for Disease and Control and Prevention’s (CDC) BRFSS. The BRFSS is a survey which collects uniform information on lifestyle behaviors, chronic health conditions, and use of medical services at the state level. State health departments manage the survey administration and report results to the CDC for data processing and analysis. Interviews are conducted via landline and cellphone calls through a random-digit-dialing technique. Eligible participants include adults aged 18 and older who have a working telephone. Each state uses a core set of questions. Many of these questions are standard each year, while others rotate every other year. Additionally, states may elect to add optional modules to their interviews which include a set of standardized questions on specific health topics. The following variables come from the 2019 BRFSS survey. More information on BRFSS data collection and sampling methods can be found on the CDC’s website (Centers for Disease Control and Prevention. Overview: BRFSS, 2019). The Institutional Review Board of the primary authors’ institution has determined use of de-identified BRFSS data as non-human subjects’ research.

### 2.2 Measures

Fruit and Vegetable Intake. There are a total of six items as part of this core module. Respondents were asked to indicate how many times per day, per week, or per month they consumed the described food. Each item was transformed into daily intake using the calculated variables provided by BRFSS. For fruit intake, there were two items (“Not including juices, how often did you eat fruit?” and “Not including fruit-flavored drinks or fruit juices with added sugar, how often did you drink 100% fruit juice such as apple or orange juice?”). A total fruit item was created by summing the responses from the two fruit items. Values over 16 were not included in the analysis as per BRFSS’s out of range value recommendation.

For vegetable intake, we examined four items (“How often did you eat a green leafy or lettuce salad, with or without other vegetables?,” “How often did you eat any kind of fried potatoes, including French fries, home fries, or hash browns?,” “How often did you eat any other kind of potatoes, or sweet potatoes, such as baked, boiled, mashed potatoes, or potato salad?,” and “Not including lettuce salads and potatoes, how often did you eat other vegetables?). A total vegetable item was created by summing the responses from the four vegetable items. Values over 23 were considered as out of range and excluded from analysis.

Adverse Childhood Experiences. There are a total of eleven questions originating from the CDC-Kaiser ACE study. The eleven questions encompass eight domains of adverse experiences including emotional abuse, sexual abuse, physical abuse, parental separation/divorce, witnessing intimate partner violence, incarcerated household member, substance abuse in the household, and mental illness in the household. Responses were dichotomized and summed into an index score ranging from 0-to-8 ACEs, with higher scores representing higher adversity. Twenty-two states added this optional module in 2019, with a total sample of 106,967 participants.

Mental Health. Mental health was assessed by the question of “Now thinking about your mental health, which includes stress, depression, and problems with emotions, how many days during the past 30 days was your mental health not good?” with responses from 0 (no days) to 30 (all days).

Physical Activity and Body Mass Index. Aerobic physical activity (PA) was categorized as meeting 150 or more minutes of PA per week (1), meeting 1–149 min of PA per week (2), and engaging in no PA throughout the week (3). Body mass index was calculated by dividing body weight in kilograms by height in meters squared.

Person-Level Characteristics. Age, sex, income, education, and race/ethnicity were included in the analysis. Age was coded in years. Sex options were male (0) and female (1). Income encompassed eight categories ranging from less than $10,000 (1) to over $75,000 (8). Education was defined by four categories including less than a high school diploma (1), completed high school diploma (2), some college (3), and completed college degree (4). Race was dummy coded with White serving as the reference group and Black/African American, Asian, American Indian/American Native, Native Hawaiian/Pacific Islander, other, multiracial, and Hispanic as comparison groups.

### 2.3 Statistical analyses

All analyses were completed using SPSS v28.0 (IBM, 2021). Histograms were constructed to assess the distribution of variables and to identify any outliers. Descriptive statistics and frequency tables were calculated for all variables. Missing data was recoded as missing. Simultaneous multiple linear regression assessed the relationship between ACEs and FVI controlling for mental health, age, sex, income, education, race/ethnicity, physical activity, and body mass index. We ran a model for each four vegetable items, both two fruit items, total fruit, and total vegetables, equaling a total of eight models. All models had the same set of covariates. Statistical significance was set with a p-value of less than 0.05.

## 3 Results

Table 1 displays the sample demographics for participants who completed the optional ACEs module. The results are stratified by those who reported to have no ACEs and by those who reported to have at least one or more ACEs. Of the total sample, nearly two-thirds (61%) reported to have at least one or more ACEs. The mean age of the total sample was 56.66 years (SD = 17.29), and 59,621 (55.7%) were female. Most of the respondents were non-Hispanic White (79.5%). Around one-third of the sample were college graduates (35.5%) and reported an annual income of over $75,000 (32.4%). Over half of the participants (50.7%) reported to engage in 150 min or more of physical activity per week. The mean body mass index was 28.70 kg/m^2^ (SD = 6.56), and the average days of poor mental health reported was 4.07 (SD = 8.35). Poor mental health days were on average higher for those who reported one or more ACEs (5.23 days, SD = 9.23) compared to those who reported no ACEs (2.21 days, SD = 6.28).

**Table 1:** Sample demographics for adults who completed ACEs module, BRFSS 2019.

	Overall (n = 106,967)	0 ACEs (n = 41,283)	≥1 ACEs (n = 65, 684)
Age in years, mean (s.d)	56.66 (17.29)	60.86 (16.33)	54.03 (17.35)
Sex, n (%)			
Female	59,621 (55.7)	22,760 (55.1)	36,861 (56.1)
Male	47,346 (44.3)	18,523 (44.9)	28,823 (43.9)
Race/ethnicity, N (%)			
White, non-Hispanic	83,561 (79.5)	33,594 (83)	49,967 (77.3)
Black, non-Hispanic	10,801 (10.3)	3,444 (8.5)	7,357 (11.4)
Asian, non-Hispanic	932 (0.9)	493 (1.2)	439 (0.7)
American Indiana/Alaska Native, non-Hispanic	1,391 (1.3)	368 (0.9)	1,023 (1.6)
Native Hawaiian/Pacific Islander, non-Hispanic	90 (0.1)	30 (0.1)	60 (0.1)
Other, non-Hispanic	714 (0.7)	250 (0.6)	464 (0.7)
Multiracial	1,790 (1.7)	408 (1.0)	1,382 (2.1)
Hispanic	5,821 (5.5)	1,905 (4.7)	3,916 (6.1)
Education, N (%)			
Some high school or less	8,109 (7.6)	2,795 (6.8)	5,314 (8.1)
High school graduate or GED	30,544 (28.6)	11,681 (28.4)	18,863 (28.8)
Some college or technical school	30,132 (28.3)	10,525 (25.6)	19,607 (29.9)
College 4 years or more (College Graduate)	37,875 (35.5)	16,127 (39.2)	21,748 (33.2)
Income (annual), N (%)			
Less than $10,000	4,142 (4.6)	1,219 (3.7)	2,923 (5.2)
$10,000 to $15,000	4,548 (5.1)	1,374 (4.2)	3,174 (5.6)
$15,000 to $20,000	6,532 (7.3)	2,119 (6.4)	4,413 (7.8)
$20,000 to $25,000	8,325 (9.3)	2,884 (8.7)	5,441 (9.7)
$25,000 to $35,000	9,618 (10.8)	3,491 (10.5)	6,127 (10.9)
$35,000 to $50,000	12,840 (14.4)	4,806 (14.5)	8,034 (14.3)
$50,000 to $75,000	14,432 (16.2)	5,447 (16.5)	8,985 (16)
More than $75,000	28,921 (32.4)	11,758 (35.5)	17,163 (30.5)
Body Mass Index, mean (s.d)	28.70 (6.56)	28.18 (6.02)	29.03 (6.86)
Poor Mental Health Days	4.07 (8.35)	2.21 (6.28)	5.23 (9.23)
150 Minutes of PA (%)			
150 + minutes of weekly PA	51,023 (50.7)	20,057 (52)	30,966 (49.9)
1-149 min of weekly PA	18,274 (18.2)	6,636 (17.2)	11,638 (18.8)
0 min of weekly PA	31,375 (31.2)	11,910 (30.9)	19,465 (31.4)
Leafy Greens per day, mean (s.d)	0.53 (0.70)	0.55 (0.69)	0.52 (0.71)
Fried Potatoes per day	0.22 (0.39)	0.20 (0.36)	0.23 (0.40)
Other Potatoes per day	0.25 (0.44)	0.25 (0.43)	0.25 (0.44)
Other Vegetables per day	0.90 (0.88)	0.90 (0.86)	0.90 (0.89)
Total Vegetables per day	1.96 (2.32)	1.96 (2.26)	1.95 (2.35)
Fruit per day	1.04 (1.01)	1.10 (1.01)	1.01 (1.02)
Fruit Juice per day	0.31 (0.63)	0.32 (0.63)	0.30 (0.64)
Total Fruit per day	1.40 (1.83)	1.47 (1.97)	1.35 (1.73)

Table 2 displays the multiple linear regression models for the four individual vegetable items and the total vegetable item. Results suggests for every one-standard unit increase in ACEs, there is a 0.020 standard deviation increase in total vegetable consumption (p <.001), holding all other variables in the model constant. In this sample, higher ACEs led to higher vegetable consumption. Upon sub-item regression analyses, we find there to be significant, positive relationships between ACEs and fried potatoes (β = 0.008), potatoes (β = 0.008), and other vegetables (β = 0.020) but not with leafy greens.

**Table 2:** Linear Regression Coefficients Predicting the Association between Total ACEs and Vegetable Intake in U.S. Adults - Adjusting for Selected Covariates, BRFSS 2019.

	Leafy Greens		Fried Potatoes		Other Potatoes		Other Vegetables		Total Vegetables	
	β	Sig.	β	Sig.	β	Sig.	β	Sig.	β	Sig.
ACE Total	.006	.119	**.008**	**.025**	**.008**	**.049**	**.024**	**.000**	**.020**	**.000**
Poor Mental Health Days	**-.026**	**.000**	**.020**	**.000**	.007	.078	**-.027**	**.000**	**-.023**	**.000**
Age	**.032**	**.000**	**-.117**	**.000**	**.036**	**.000**	**-.010**	**.006**	**-.010**	**.012**
Sex	**.068**	**.000**	**-.124**	**.000**	**-.032**	**.000**	**.122**	**.000**	**.069**	**.000**
Body Mass Index	**-.011**	**.002**	.006	.109	**-.034**	**.000**	-.005	.147	**-.017**	**.000**
150 Minutes of PA	**-.104**	**.000**	**.035**	**.000**	-.002	.613	**-.094**	**.000**	**-.100**	**.000**
Education	**.065**	**.000**	**-.063**	**.000**	**-.038**	**.000**	**.070**	**.000**	**.047**	**.000**
Income	**.060**	**.000**	**-.010**	**.015**	**-.029**	**.000**	**.041**	**.000**	**.045**	**.000**
Race - Black	**.010**	**.008**	-.002	.604	**-.040**	**.000**	**-.021**	**.000**	**-.021**	**.000**
Race - AI/AN	**.016**	**.000**	**.012**	**.001**	**.010**	**.006**	-.000	.932	**.016**	**.000**
Race - Asian	**.019**	**.000**	**-.022**	**.000**	**-.016**	**.000**	**.012**	**.001**	.007	.057
Race - Nat. Haw. Pac Is	.005	.187	**.011**	**.001**	-.005	.142	-.002	.574	.003	.403
Race - Other	**.015**	**.000**	.000	.926	-.004	.270	.003	.470	**.007**	**.043**
Race - Multiracial	**.013**	**.000**	-.004	.229	**.009**	**.014**	**.014**	**.000**	**.018**	**.000**
Hispanic	**.025**	**.000**	**-.017**	**.000**	**-.036**	**.000**	**-.036**	**.000**	**-.024**	**.000**
F Statistic	**182.240**		**208.107**		**50.698**		**220.293**		**150.706**	
R^2^	.034		.038		.010		.040		0.029	

Note: Boldface indicates statistical significance (p < 0.05) Coefficients are standardized.

Table 3 displays the multiple linear regression models for the two individual fruit items and the total fruit item. Results suggests that for every one-standard deviation unit increase in ACEs, there is a 0.015 standard deviation decrease in total fruit consumption (p <.001), holding all other variables in the model constant. In this sample, higher ACEs led to lower fruit consumption. Upon sub-item regression analyses, canned, fresh, or frozen fruit is driving the relationship between ACEs and decreased fruit intake (β =  − 0.016), whereas there is a nonsignificant relationship between ACEs and fruit juice.

**Table 3:** Linear Regression Coefficients Predicting the Association between Total ACEs and Fruit Intake in U.S. Adults - Adjusting for Selected Covariates, BRFSS 2019.

	Fruit		Fruit Juice		Total Fruit	
	β	Sig.	β	Sig.	β	Sig.
ACE Total	**-0.016**	**0.000**	-0.001	0.757	**-0.015**	**0.000**
Poor Mental Health Days	**-0.032**	**0.000**	0.002	0.642	**-0.025**	**0.000**
Age	**0.041**	**0.000**	**0.046**	**0.000**	**0.056**	**0.000**
Sex	**0.098**	**0.000**	**-0.074**	**0.000**	**0.044**	**0.000**
Body Mass Index	**-0.026**	**0.000**	**-0.035**	**0.000**	**-0.038**	**0.000**
150 Minutes of PA	**-0.111**	**0.000**	**-0.031**	**0.000**	**-0.105**	**0.000**
Education	**0.073**	**0.000**	-0.005	0.214	**0.057**	**0.000**
Income	**0.032**	**0.000**	**-0.067**	**0.000**	-0.006	0.171
Race - Black	0.003	0.446	**0.085**	**0.000**	**0.045**	**0.000**
Race - AI/AN	**0.013**	**0.000**	**0.022**	**0.000**	**0.022**	**0.000**
Race - Asian	**0.018**	**0.000**	-0.005	0.169	**0.013**	**0.000**
Race - Nat. Haw. Pac Is	0.004	0.255	0.000	0.981	0.004	0.311
Race - Other	**0.007**	**0.043**	**0.010**	**0.005**	**0.011**	**0.002**
Race - Multiracial	**0.016**	**0.000**	**0.027**	**0.000**	**0.026**	**0.000**
Hispanic	**0.026**	**0.000**	**0.031**	**0.000**	**0.036**	**0.000**
F Statistic	**222.530**		**114.34**		**149.361**	
R^2^	0.041		0.021		0.028	

Note: Boldface indicates statistical significance (p < 0.05) Coefficients are standardized.

The analysis reveals notable observations among person-level characteristics. For example, being a racial/ethnic minority is related to higher leafy green and canned, fresh, or frozen fruit consumption, holding all else in the model constant. Yet, these results are mixed with regards to the other vegetable and fruit items. For instance, compared to non-Hispanic whites, Hispanics scored (β = 0.025) higher on leafy green consumption, but scored lower on total vegetable consumption (β = -0.024).

## 4 Discussion

The current study’s goal was to examine the relationship between total ACEs and FVI among adults. Regression analyses were performed to identify the direction of the relationship between total ACEs and total FVI as well as which specific fruit and vegetable items drove these findings. Higher total ACE scores were associated with lower consumption of fruit. Specifically, fresh, frozen, and canned fruit consumption decreased as ACEs increased. Since fruits are an important food group in a nutrient-dense eating pattern, chronic underconsumption of fruit could predispose people to malnutrition.

Unlike fruit, as the total number of ACEs increased so did vegetable intake. Fried potatoes, potatoes, and other vegetables had significant relationships with ACEs, whereas leafy greens did not. Fried potatoes are a high calorie-dense vegetable option and are often prepared by adding dietary fat and sodium in the cooking process (U.S. Department of Agriculture and National Institutes of Health, 2020). This study supports as ACE scores increase, so does fried potato consumption. Trauma stimulates higher rates of emotional dysregulation which is related to emotional eating (Talbot et al., 2013, Guerrini-Usubini et al., 2023, Michopoulos et al., 2015). Thus, it is possible that people with higher ACEs use emotional eating to respond to trauma-induced toxic stress. Emotional eating has been related to higher consumption of energy-dense foods, (Camilleri et al., 2014) which may explain the positive relationship between ACEs and fried potato consumption.

There were unexpected, positive relationships with ACEs and non-fried potatoes. One potential explanation for these findings is the BRFSS questionnaire did not capture the context in which all food items were consumed and prepared. For instance, potatoes dishes that are unfried may still be high in calories, dietary fat, and sodium. Mashed potatoes, stuffed baked potatoes, and potato casseroles are examples of potato-based dishes often prepared with whole fat dairy or protein products. Foods prepared in their energy-dense form can be referred to as comfort food. Prior evidence supports that chronic stress can promote greater consumption of comfort food, (Dallman et al., 2003, Gibson, 2012) which may explain why higher ACEs were associated with higher non-fried potato intake. Further analysis with an in-depth dietary analysis considering food preparation should take place to confirm this. The importance of context can also be demonstrated in the findings between ACEs and other vegetables. This item consists of raw, cooked, canned, and frozen vegetables, but it does not ask how vegetables were prepared. Differences in cooking methods can change the nutritive value of vegetables, (Lee et al., 2018) or it can add extra calories from fat (Raber et al., 2020). Similar to non-fried potatoes, higher ACEs could relate to higher other vegetable intake because of the methods people with ACEs prepared the vegetables (e.g., in comfort food form) in response to toxic stress.

Fruit findings are consistent with other studies, but the vegetable findings are mixed. Horino and Yang (2021) using Nevada BRFSS data found individuals with three or more ACEs had significantly higher odds of having low FVI. In their study, investigators defined low FVI as eating fruits and vegetables less than 2 times a day and only used the fruit, dark leafy green, and other vegetable items in analysis. Another study (Schuler et al., 2021) pulling data from the Early Childhood Longitudinal Study-Birth Cohort found there to be significant decreased fruit intake particularly in those who experienced domestic violence or had a family member incarcerated. The same study found non-significant, and in many cases, positive vegetable findings across ACE modules when excluding fried potatoes.

This study yielded unique findings regarding person-level characteristics and FVI. Why racial/ethnic minority status resulted in higher leafy green consumption is unclear. Prevalence data from the 2017–2018 What We Eat in America (WWEIA) survey reported Hispanics to report consuming vegetables at the lowest frequency (Hoy et al., 2021). The current study supports that Hispanics, compared to non-Hispanic Whites, have the largest, negative effect size in total vegetable intake (β = -0.024) relative to other racial and ethnic minorities, which supports findings from the WWEIA survey. However, compared to non-Hispanic Whites, Hispanics had the largest, positive effect size for increased leafy green consumption (β = 0.025) relative to other racial and ethnic minorities. Further research is warranted to explore subgroup fruit and vegetable intake by race/ethnicity and its impact on human health to identify and reduce diet-related health disparities (Satia, 2009).

### 4.1 Study limitations and strengths

This study has its limitations. The cross-sectional nature of this data limits its assumptions for causality and directionality. Additionally, measures in BRFSS are self-reported which opens opportunity for social desirability bias particularly for items such as FVI, (Miller et al., 2008) physical activity, (Adams et al., 2005) and BMI (Burke and Carman, 2017) among adults. While the BRFSS ACEs module serves as an important surveillance tool, it does not capture the severity of the traumatic experience which may elucidate different stress responses (Anda et al., 2020). BRFSS is a telephone-based survey that does not reach certain segments who may be predisposed to ACEs such as the criminal justice involved population or those living in poverty (Testa et al., 2021, Pew Research Center, 2022).

The BRFSS fruit and vegetable module does not approximate cup-equivalents (Moore et al., 2015). This limits the direct comparison between BRFSS data and federal nutrition standards such as the Dietary Guidelines. Based on a 2,000-calorie diet, the DGA recommends 2.5 cups equivalents of vegetables and 2 cup equivalents of fruit per day. A 1-cup equivalent is equal to 1 cup of raw or cooked fruit/vegetable, 1 cup fruit or vegetable juice, 2 cups of leafy salad greens, and ½ cup dried fruits or vegetables (U.S. Department of Agriculture and National Institutes of Health, 2020). The BRFSS questions address frequency which may be different from cup-equivalents given the differences in portion size consumption among respondents. For instance, two people could both report eating fruits twice a day, and one person could have consumed a one-cup equivalent, whereas another person could have consumed three-cup equivalents.

This study is derived from a large, national sample which provides a snapshot of health statuses and behaviors among U.S. adults. Similarly, by exploring fruit and vegetable intake separately we were able to identify which subgroups of foods drove the relationship between ACEs and total FVI. As demonstrated, certain items can inflate the relationship between ACEs and total FVI, or ACEs may not be related to certain fruit or vegetable items. This study points to specific fruit and vegetable items to illustrate unique dietary patterns among people with ACEs.

### 4.2 Future directions

Our findings underscore the need for studies which examine the contextual factors of dietary behaviors, including the preparation of fruit and vegetables, among those experiencing ACEs. This could include the use of 24-hour dietary recalls which provides more distinct data on the amount, preparation, and timing of foods (National Cancer Institute. Dietary Assessment Primer, 2022). Previous studies examining the relationship between ACEs and dietary habits suggests toxic stress is an underlying mechanism (Horino and Yang, 2021, Chiu et al., 2021, Morton et al., 2021, Jackson and Vaughn, 2019). Studies should examine whether acute negative moods or stress would heighten poor dietary habits, specifically among people with ACEs who already experience high levels of stress. Digital food diaries could be a measurement tool to document moods (e.g., anger, stress, sadness) before snack and meal consumption (Young et al., 2021). Additionally, researchers should examine specific aspects of ACEs, such as the severity or duration of the traumatic experience. While it is broadly accepted that people with ACEs experience toxic stress, (Nelson et al., 2020) identifying distinctions among experiences may support the creation of interventions.

Future studies should examine efforts to improve the social conditions and environments for those with the ACEs as it relates to the access of nutritious foods. People with ACEs experience heightened issues with food insecurity, (Testa and Jackson, 2020) which may lead to disparities in adequate fruit and vegetable intake (Howard, 2013). Exposure to ACEs is longitudinally associated with social determinants related to food insecurity (e.g., economic instability) (Bunting et al., 2018). Improving these social determinants of health will require combined efforts from multiple sectors including housing, education, transportation, and public health organizations (Office of Disease Prevention and Health Promotion. Healthy People, 2030).

The U.S. government should invest in epidemiological systems which rigorously measure both nutritional and ACE outcomes. BRFSS is among few national surveillance systems that captures both dietary behaviors and ACEs in the United States. The National Health and Nutrition Examination Survey (NHANES) is another surveillance system that measures dietary behaviors (utilizing 24-hour dietary recalls), but it does not currently measure childhood adversity. Adding an ACEs module to NHANES that measures beyond a dichotomous response may provide additional insight on how the severity or duration of an ACE could impact dietary outcomes. These surveillance systems, along with other epidemiological data, guides preventative decision making such as those set by the DGA. The guidelines set nutritional standards for federal food assistance programs such as Supplemental Nutrition Assistance Program and the National School Lunch Program. Supporting the expansions of such programs may reduce levels food insecurity (Rivera et al., 2019) and child maltreatment, (Maguire-Jack et al., 2022) while increasing FVI (Olsho et al., 2016).

## 5 Conclusion

This study found that after adjusting for person-level factors, higher ACEs were associated to lower fruit consumption and higher fried potato, non-fried potato, and other vegetable consumption. Results did not find associations between ACEs and fruit juice and leafy green vegetables. Findings emphasize the need to understand food preparation and context when examining the relationship between ACEs and FVI.

## Financial disclosure

Publication of this article was funded in part by Purdue University Libraries Open Access Publishing Fund.

## CRediT authorship contribution statement

**Ivan D. Mendoza:** Conceptualization, Formal analysis, Methodology, Writing – original draft, Writing – review & editing. **Jorge A. Banda:** Conceptualization, Methodology, Writing – original draft, Writing – review & editing. **Zachary Giano:** Conceptualization, Formal analysis, Methodology, Writing – original draft, Writing – review & editing. **Randolph D. Hubach:** Conceptualization, Methodology, Writing – original draft, Writing – review & editing.

## Declaration of Competing Interest

The authors declare that they have no known competing financial interests or personal relationships that could have appeared to influence the work reported in this paper.

## Data Availability

Data will be made available on request.
